# *In Silico* Identification of Novel Biomarkers and Development of New Rapid Diagnostic Tests for the Filarial Parasites *Mansonella perstans* and *Mansonella ozzardi*

**DOI:** 10.1038/s41598-019-46550-9

**Published:** 2019-07-16

**Authors:** C. B. Poole, A. Sinha, L. Ettwiller, L. Apone, K. McKay, V. Panchapakesa, N. F. Lima, M. U. Ferreira, S. Wanji, C. K. S. Carlow

**Affiliations:** 1 New England Biolabs, Massachusetts, USA; 2 Department of Parasitology, Institute of Biomedical Sciences, University of São Paulo, São Paulo, Brazil; 3 Department of Microbiology and Parasitology, University of Buea, Buea, Cameroon

**Keywords:** Data mining, Diagnostic markers

## Abstract

Mansonelliasis is a widespread yet neglected tropical infection of humans in Africa and South America caused by the filarial nematodes, *Mansonella perstans*, *M*. *ozzardi*, *M*. *rodhaini* and *M*. *streptocerca*. Clinical symptoms are non-distinct and diagnosis mainly relies on the detection of microfilariae in skin or blood. Species-specific DNA repeat sequences have been used as highly sensitive biomarkers for filarial nematodes. We have developed a bioinformatic pipeline to mine Illumina reads obtained from sequencing *M*. *perstans* and *M*. *ozzardi* genomic DNA for new repeat biomarker candidates which were used to develop loop-mediated isothermal amplification (LAMP) diagnostic tests. The *M*. *perstans* assay based on the Mp419 repeat has a limit of detection of 0.1 pg, equivalent of 1/1000^th^ of a microfilaria, while the *M*. *ozzardi* assay based on the Mo2 repeat can detect as little as 0.01 pg. Both LAMP tests possess remarkable species-specificity as they did not amplify non-target DNAs from closely related filarial species, human or vectors. We show that both assays perform successfully on infected human samples. Additionally, we demonstrate the suitability of Mp419 to detect *M*. *perstans* infection in *Culicoides* midges. These new tools are field deployable and suitable for the surveillance of these understudied filarial infections.

## Introduction

Mansonelliasis (or mansonellosis) is an infection of humans caused by one of four species of nematodes: *Mansonella perstans*, *M*. *ozzardi*, *M*. *streptocerca* and *M*. *rodhaini*. *M*. *perstans* has the widest distribution and is endemic in 33 countries in sub-Saharan Africa. It is also found in the northern coast of South America including Amazonian Colombia and equatorial Brazil^1,2^, following introduction into the New World through the slave trade^3^. Although the global burden of mansonelliasis is not well defined^1^, it is estimated that in Africa alone, 114 million people are infected with *M*. *perstans* with another 580 million individuals at risk of infection^2^ with prevalence rates in affected communities that range from 3–96%^4^. *M*. *ozzardi*, *M*. *streptocerca and M*. *rodhaini* have a more restricted distribution. *M*. *streptocerca* is localized to tropical rainforest areas of central and west Africa^1^ where the prevalence rates also vary widely, ranging from 0.5–89%^4^. The distribution of *M*. *ozzardi* extends from southern Mexico through parts of the Caribbean as well as Central and South America^1,5,6^ where prevalence in affected communities ranges from <1–46%^5,6^. Several dozen human infections with *M*. *rodhaini* have been observed in forested regions of Gabon^7^.

Infective third stage larvae (L3s) of *Mansonella* are transmitted to humans by biting midges of the genus *Culicoides*^2,6–9^. *M*. *ozzardi* L3s are also transmitted by biting black flies of the genus *Simulium* in Central and South America^6^. Adult male and female worms mate in the human host and females produce viviparous unsheathed microfilariae (mf) that circulate in the blood (*M*. *perstans*^2^ and *M*. *ozzardi*^6^) or skin (*M*. *ozzardi*^6^, *M*. *streptocerca*^10^*, M*. *rodhaini*^7,11^). Following ingestion by an arthropod vector during a blood meal, mf develop into infective L3s that are transmitted to a human host in the course of a subsequent feeding.

Mansonelliasis is the least studied of the filarial diseases as *Mansonella* spp. parasites generally cause less pathology than other filarial worms. Infected individuals commonly experience an array of non-specific symptoms and signs including fever, headache, joint pain, lymphadenopathy, chronic pruritis, rashes and other dermatological symptoms^2,5,6,12^. In addition, some patients suffer from ocular lesions due to the presence of adult *M*. *perstans*^13,14^ or *M*. *ozzardi* mf^5,15^ in the eyes. Mansonelliasis, particularly *M*. *perstans* infections may also affect people in more subtle ways such as the down regulation of immune responses^16^ enhancing susceptibility to other infections as well as decreasing the efficacy of vaccine programs^1^.

*Mansonella* spp. exhibit significant differences in their susceptibility to the common chemotherapeutics used for treatment of filarial infections. *M*. *perstans* infections are considered the most difficult to treat as this parasite is unresponsive to ivermectin^17,18^; however, a combination of diethylcarbamazine (DEC) and mebendazole has proved effective^2,18^. In contrast, a single dose of ivermectin will completely clear *M*. *ozzardi* mf for at least a year^19^. Although the use of DEC^1,12^ or ivermectin^12,20^ have proved efficacious for *M*. *streptocerca* infections, ivermectin is preferred in areas co-endemic with onchocerciasis due to the adverse side effects of DEC^20^. Doxycycline has been used to treat infections caused by filarial parasites which harbor the bacterial endosymbiont *Wolbachia*^21,22^ including *M*. *perstans*^23^. The susceptability of *M*. *rodhaini* to these drugs is not known.

Given the largely non-specific clinical symptoms presented in mansonelliasis and the difficulty of detecting adult parasites^5,24^, definitive diagnosis rests on detection of mf in skin or blood samples^25–28^. In addition to the commonly used thick blood smear method, concentration techniques including Knott’s method or polycarbonate membrane filtration may be employed for low-density microfilaremias^5^. Differentiation of mf is based on the absence of a sheath, the staining pattern of terminal nuclei in the tail as well as its shape. *M*. *steptocerca* are readily distinguished from other mf by their hooked-shaped tails, whereas *M*. *rodhaini* mf are distinguished by their long length^24^. *M*. *perstans* have blunt rounded tails and the tails of *M*. *ozzardi* taper to a point^1,29^. While microscopy is a valuable technique, morphological interpretation can be subjective and requires substantial expertise. Currently, no specific immunodiagnostic tests are available for *Mansonella* infection^1^ and molecular diagnosis relies on polymerase chain reaction (PCR) amplification of DNA targets. Most assays target the internal transcribed spacer (ITS) regions between the conserved ribosomal genes 18S, 5.8S and 28S,^30–34^ or the spacer region between 5S rDNA genes^10^. The majority of these assays distinguish either *M*. *perstans* or *M*. *ozzardi* from sympatric filarial parasites of other genera^30,31,33,35–37^ but do not differentiate *M*. *perstans* from *M*. *ozzardi*. A PCR assay has been developed to differentiate *M*. *perstans* from *M*. *ozzardi*, however, this requires sequencing of the amplicon^30^. In general, these molecular assays are more sensitive than the standard parasitological techniques for the identification of *Mansonella spp*.^1,10,31–34,38–40^. Nevertheless, their dependence on trained personnel and relatively expensive equipment restricts their widespread adoption^41,42^. In addition, PCR reactions require considerable time from DNA extraction to visual read-out by gel electrophoresis or other confirmatory techniques and as such are not appropriate when rapid diagnoses are needed. Therefore, despite their performance, PCR techniques are mostly limited to research facilities and are not ideally suited to field conditions or low-resource settings.

Several isothermal amplification methods targeting DNA have been developed which offer significant improvements over PCR^43^. Of these methods, loop-mediated isothermal amplification (LAMP) has become the most widely adopted. Its simplicity and visual detection format without the need for expensive equipment offers considerable advantages over PCR^41,42,44,45^. LAMP assays displaying high levels of specificity and sensitivity have been described for several filarial nematodes including *Brugia malayi*^46^, *Brugia timori*^46^, *Loa loa*^47–49^, *Onchocerca volvulus*^50–52^, *Wuchereria bancrofti*^45,53^, *Dirofilaria immitis*^54^ and *Dirofilaria repens*^55^. Genome sequencing has brought with it the ability to devise comparative and subtractive strategies to identify, *in silico*, new diagnostic candidates from an organism’s genome^47^. Repeat sequences, some of which occur at high copy number in nematode genomes, are particularly attractive targets as biomarkers^45–47,56,57^. However, the assembly of repeat regions remains a challenge for shotgun sequencing methods, particularly with short-read technologies like Illumina. To identify repeat-based biomarkers for *M*. *perstans* and *M*. *ozzardi*, while circumventing the requirement of a high-quality genome assembly, we developed a novel bioinformatic strategy to directly mine for repeat sequences from raw sequencing reads. The candidate biomarkers discovered were validated experimentally and used to develop new rapid colorimetric LAMP assays. These assays were evaluated on patient samples and infected midges and compared with microscopy and PCR-based methods.

## Results

### Bioinformatic identification of diagnostic candidates

**Figure 1:**
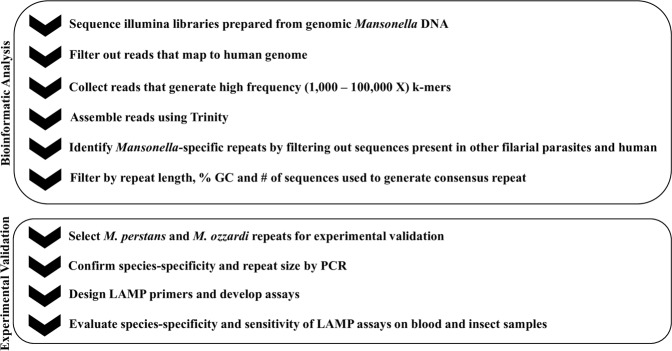
Bioinformatic and experimental pipeline to identify *M*. *perstans* and *M*. *ozzardi* specific repeats. Consensus repeat sequences that are at least 300 bp in length were ranked according to %GC. Candidate repeat sequences were evaluated for species-specificity and sensitivity using PCR, and the most promising candidate for each species was selected for LAMP assay development.

To enable the identification of new species-specific DNA biomarkers for *M*. *perstans* and *M*. *ozzardi* using a subtractive genomic filtering approach (Fig. 1), Illumina libraries were constructed and sequenced. The total number of reads obtained from the *M*. *perstans* and *M*. *ozzardi* libraries were ~227 × 10^6^ and ~279 × 10^6^, respectively. Contaminating human reads present in the *M*. *perstans* (10%) and *M*. *ozzardi* (94%) libraries were removed, and high frequency k-mer reads collected from each library (*M*. *perstans*, 2,077,887; *M*. *ozzardi*, 138,139) were assembled to generate 61,182 candidate repeat sequences for *M*. *perstans* and 47 for *M*. *ozzardi*. After removing homologous sequences present in the genomes of human and other filarial parasites, 735 *Mansonella* spp. specific candidate repeats were identified using data derived from the *M*. *perstans* library. Of the 47 candidate repeat sequences from *M*. *ozzardi*, 5 were predicted to be species-specific.

Target copy number is an important consideration for biomarker discovery as it may influence assay sensitivity^57–59^. However, since raw reads were used and accurate copy number data was not available, the number of aligned sequencing reads (9,269–119,769) used to generate each consensus repeat sequence was used as a proxy. The consensus repeats identified were then ranked by size (>300 bps) and % GC (29–42) content to facilitate LAMP primer design. A total of 109 *M*. *perstans* and one *M*. *ozzardi* repeat families were identified that fulfilled the selection criteria for new candidate DNA biomarkers suitable for LAMP assay development. Of these, 16 *M*. *perstans* and one *M*. *ozzardi* consensus repeats were chosen for further evaluation. Additional characterization by blastn identified the presence of internal repeats in 7 of 17 *Mansonella* consensus sequences. When primers were designed to each consensus repeat sequence and assayed by PCR to confirm the presence of these sequences in DNA, those containing internal repeats displayed a ladder-like banding pattern on a gel, with steps ranging from ~50–180 bps in size, indicative of a tandem organization in the genome. Whereas the remaining primer sets amplified single bands of the expected MW, suggestive of dispersed repeats. Additional analysis revealed that the *M*. *perstans* candidates collapsed into 10 unique consensus sequences. Of these, 2 dispersed (Mp396, Mp415) and 2 tandem (Mp419, Mp347) repeats (containing a minimum repeat size of 180 bp) as well the *M*. *ozzardi* tandemly arranged repeat (Mo2) were selected for LAMP primer design, assay development and optimization.

### Evaluation of diagnostic candidates

**Figure 2:**
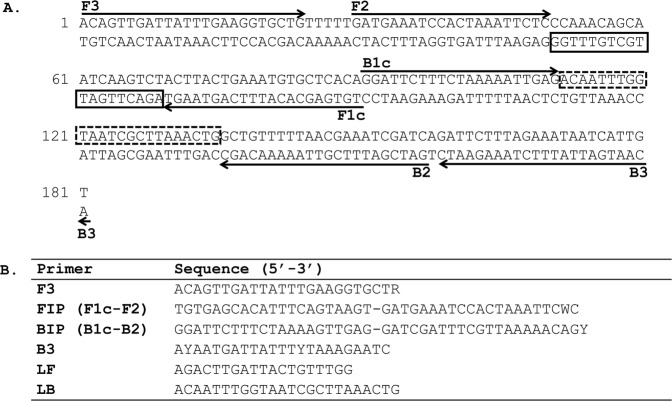
LAMP primer set targeting the *M*. *perstans* Mp419 repeat. (**A**) The location of the six LAMP primers within the consensus sequence is shown. Arrows indicate the direction of extension. The solid and dash line boxes represent the binding regions of the loop forward (LF) and loop back (LB) primers respectively. (**B**) Sequence of the LAMP primers. F3, FIP, BIP and B3 are degenerate primers where R = A or G, W = A or T and Y = C or T.

**Figure 3:**
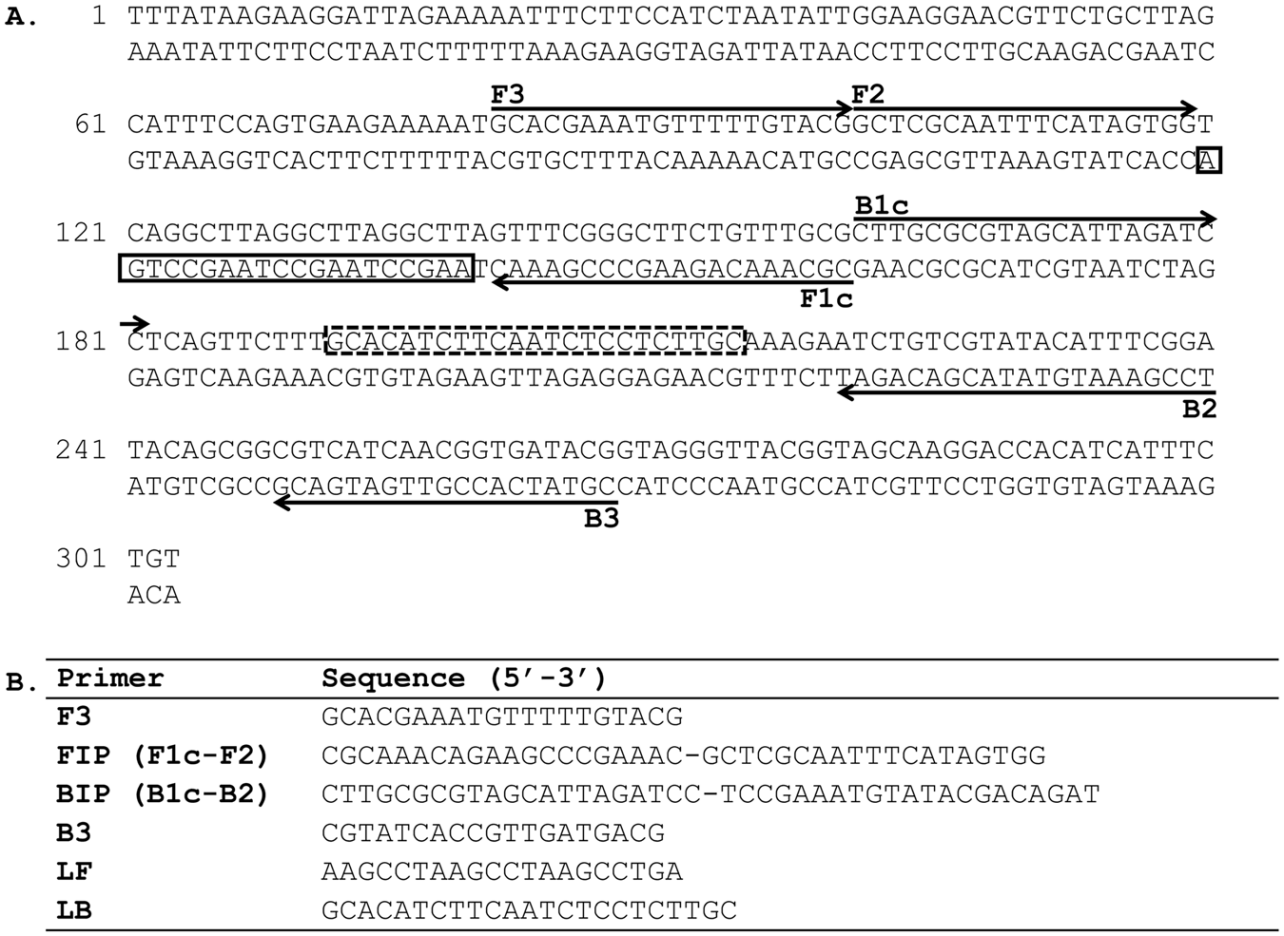
LAMP primer set targeting the *M*. *ozzardi* Mo2 repeat. (**A**) The location of the six LAMP primers within the consensus sequence is shown. Arrows indicate the direction of extension. The solid and dash line boxes represent the binding regions of the loop forward (LF) and loop back (LB) primers respectively. (**B**) Sequence of the LAMP primers.

**Figure 4:**
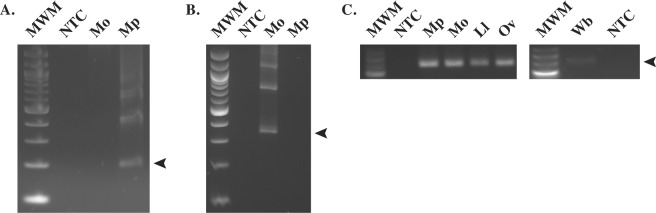
Genomic organization of the *Mansonella* target repeats. Agarose gels showing PCR amplification of the *M*. *perstans* (**A**) and *M*. *ozzardi* (**B**) repeats. A 100 bp DNA ladder (New England Biolabs) was used as the molecular weight marker (MWM). Arrows denote the monomer band of each ladder (*M*. *perstans*, ~200 bp; *M*. *ozzardi*, ~300 bp). As a positive control for the presence of DNA (**C**), a conserved 244 bp actin fragment was PCR amplified from *M*. *perstans* (Mp), *M*. *ozzardi* (Mo), *L*. *loa* (Ll), *O*. *volvulus* (Ov) or *W*. *bancrofti* (Wb) DNA. Full-length gels for (**C**) are presented in Supplementary Fig. 4. The low molecular weight DNA ladder (New England Biolabs) was used as the molecular weight marker (MWM). Water was substituted for DNA in the non-template controls (NTC).

Multiple primer sets corresponding to the consensus sequence generated for each repeat family were designed and tested empirically in LAMP amplification assays. All repeat families except *M*. *perstans* Mp419 (Fig. 2) and *M*. *ozzardi* Mo2 (Fig. 3), were eventually eliminated from consideration (data not shown) due to either poor sensitivity or high non-specific background in LAMP reactions. The consensus sequence for Mp419 is 181 bp in length and 33% GC rich. PCR amplification of Mp419 produces a DNA ladder with steps ~200 bps apart, confirming the tandem arrangement of this repeat family in genomic DNA (Fig. 4A). Mo2 is 303 bp in length and 42% GC rich. PCR amplification of Mo2 also revealed a ladder array, with steps ~300 bps apart (Fig. 4B).

### Specificity of the *Mansonella* biomarkers in DNA amplification assays

PCR experiments confirmed the species-specificity of Mp419 (Fig. 4A) and Mo2 (Fig. 4B) where it was observed that primers targeting their respective repeats did not amplify DNA from the other *Mansonella* species. The non-template controls (NTC) in each experiment were negative. Furthermore, no amplification was observed using *L*. *loa*, *W*. *bancrofti*, *O*. *volvulus*, *C*. *sonorensis*, or human DNA (data not shown). The integrity of the *Mansonella* DNA samples was confirmed in PCR experiments using primers designed to amplify a conserved actin gene^46^. A single amplification product of 244 bp, the expected fragment size was obtained (Fig. 4C).

**Figure 5:**
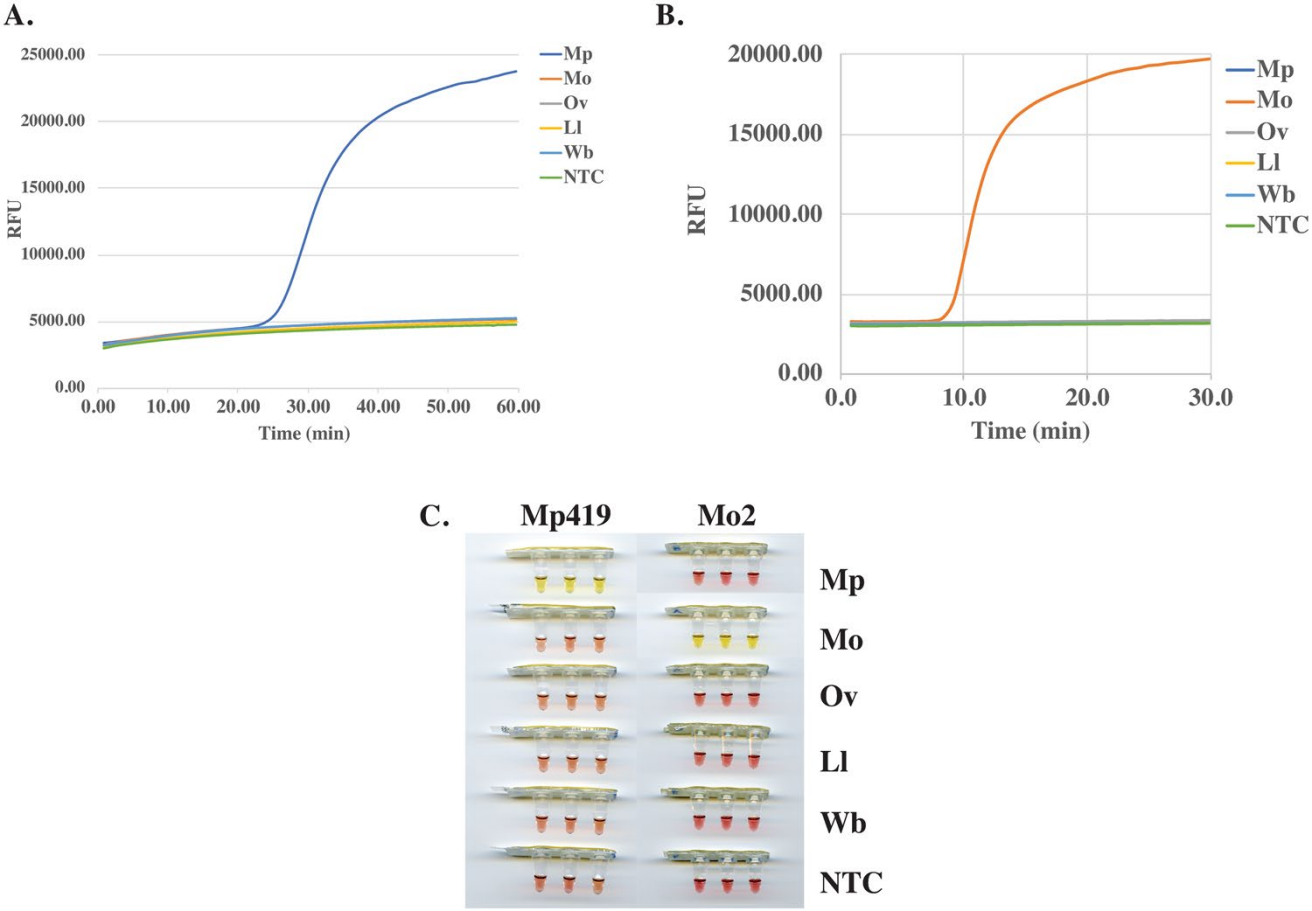
Species-specificity of the *Mansonella* LAMP assays. Reactions containing DNA (100 pg) from *M*. *perstans* (Mp), *M*. *ozzardi* (Mo), *O*. *volvulus* (Ov), *L*. *loa* (Ll) or *W*. *bancrofti* (Wb), and either the *M*. *perstans* or *M*. *ozzardi* LAMP primers were performed in colorimetric master mix containing a fluorescent dsDNA binding dye. DNA amplification was monitored in real time using a qPCR machine. The real time amplification curves for the Mp419 (**A**) and Mo2 (**B**), as well as the end-point color change of the LAMP reactions (**C**) are shown. Water was substituted for DNA in the non-template controls (NTC).

The 181 bp Mp419 sequence (Fig. 2A) necessitated that the LAMP primer set be designed manually whereas the longer 303 bp Mo2 sequence (Fig. 3A) was submitted to Primer Explorer for LAMP primer design. The species-specificity of the primer sets were evaluated in colorimetric LAMP reactions containing a fluorescent dye to enable reaction dynamics to be monitored in real time. The *M*. *perstans* primer set only amplified *M*. *perstans* DNA (Fig. 5A). Amplification was evident at ~25 min and continued until the reactions were terminated at 60 min. Species-specific amplification was also observed with the *M*. *ozzardi* primer set at ~8 min and continued until reaction termination at 30 min (Fig. 5B). Neither primer set amplified the non-targeted *Mansonella* species nor *O*. *volvulus*, *L*. *loa* or *W*. *bancrofti* DNA (Fig. 5A,B). In addition, no amplification was observed in the NTCs by either primer set. The end point color change in these reactions are consistent with the results obtained using the fluorescent dye (Fig. 5C). The integrity of the *L*. *loa, O*. *volvulus* and *W*. *bancrofti* DNA samples (Fig. 4C) was confirmed using an actin amplicon as described above.

### Analytical sensitivity of the *Mansonella* LAMP assays

**Figure 6:**
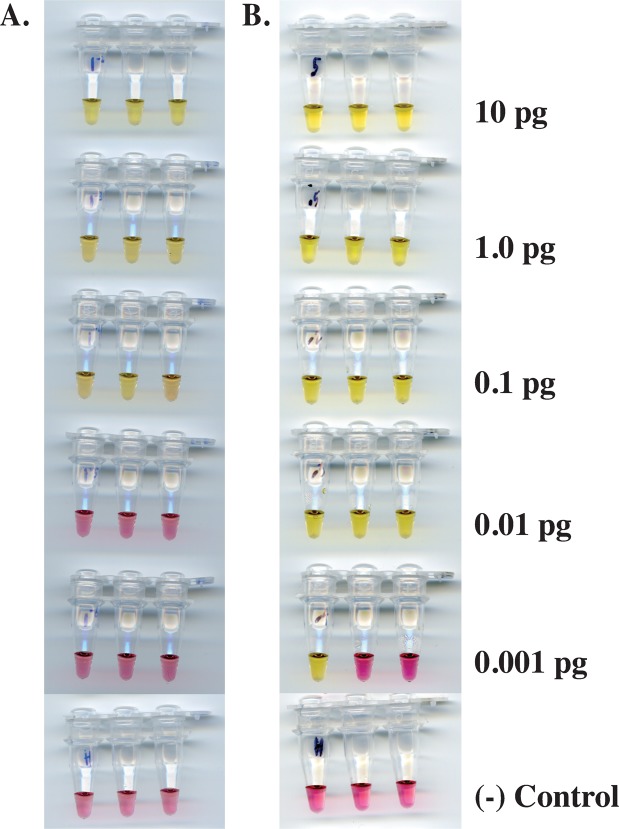
Sensitivity of the *Mansonella* LAMP assays. DNA from *M*. *perstans* (**A**) or *M*. *ozzardi* (**B**) ranging from 10–0.001 pg per reaction, was amplified using the primer sets targeting Mp419 or Mo2, respectively. Negative (−) controls containing 1 ng of HeLa DNA (New England Biolabs) were included.

The sensitivity of the *Mansonella* LAMP assays was evaluated using a 10X dilution series ranging from 10–0.001 pg of genomic DNA. After a 60 min incubation, as little as 0.1 pg of *M*. *perstans* DNA was detected, as indicated by the color change from pink to yellow. No color change was observed in either the 0.01 or 0.001 pg samples (Fig. 6A). A similar level of sensitivity was observed (Supplementary Fig. 1) using a previously published, but complicated, nested-PCR assay^31^. The *M*. *ozzardi* assay has a limit of detection of 0.01 pg in 30 min (Fig. 6B). No color change was observed in samples containing only human DNA.

### Evaluation of the *Mansonella* LAMP assays on insect and blood samples

Diagnostic assays based on the amplification of pathogen DNA can be used to detect infection in both vectors and humans, therefore, the performance of the *Mansonella* LAMP assays was evaluated on experimentally infected midges and clinically defined blood samples. DNA was extracted from 36 *Culicoides milnei* which had fed on a *M*. *perstans* infected volunteer^9^. As negative controls, DNA was also prepared from 36 unfed *C*. *milnei* midges. Each insect sample was evaluated using the colorimetric Mp419 LAMP assay as well as a previously published ITS1-based nested-PCR assay^31^ (Table 1). Of the 36 fed midges, 10 scored positive by *M*. *perstans* LAMP while 14 scored positive in nested-PCR. The flies which were positive in LAMP were also positive in nested-PCR. None of the 36 unfed midges scored positive by LAMP. The nested-PCR product generated in the 4 midge samples which were negative by LAMP were sequenced and found to originate from *M*. *perstans* (Supplementary Fig. 2). No amplification was observed in the NTCs in either LAMP or nested-PCR assays. Based on these data (Table 1), the sensitivity of the LAMP assay was determined to be 71.4% while specificity was determined to 100%. The 95% confidence interval (CI) for sensitivity was calculated to range from 43.4% to 99.4%.

**Table 1:** Comparison of ITS1 nested-PCR and colorimetric Mp419 LAMP assays for detection of *M*. *perstans* in *C*. *milnei*.

	Nested PCR Positive	Nested PCR Negative
LAMP Positive	10	0
LAMP Negative	4	58

To evaluate the performance of the *M*. *perstans* LAMP assay, DNA was prepared from human samples collected from a small number of individuals (n = 10) in Cameroon, and non-endemic negative controls from either Brazil (n = 5) or the United States (n = 5). All 20 samples were evaluated by nested-PCR and microscopy. All 10 endemic samples from Cameroon scored positive by LAMP and nested-PCR (Table 2), while nine of these 10 scored positive by microscopy (Supplementary Fig. 3). NTCs in both LAMP and nested-PCR assays were negative. To determine sensitivity and specificity using the CRS test, results from microscopy and nested-PCR were designated as the imperfect gold standard and imperfect resolver respectively (Supplementary Fig. 3). The sensitivity and specificity were both calculated to be 100% (Table 2).

**Table 2:** Evaluation of Mp419 LAMP for detection of *M*. *perstans* in patient samples using the CRS test.

	Nested PCR Positive	Nested PCR Negative
LAMP Positive	10	0
LAMP Negative	0	10

The *M*. *ozzardi* LAMP assay was evaluated using 83 samples from Brazilian individuals whose infection status regarding *M*. *ozzardi* was previously determined by microscopy or qPCR using an ITS-2 based assay^60^. All 51 patients that were reported to be *M*. *ozzardi* positive^60^ scored positive in the Mo2 LAMP assay (Table 3). Of the 33 samples that were reported to be negative^60^, 8 scored positive in the Mo2 LAMP assay as well as in the ITS1-nested PCR assay (Table 3). The remaining 25 samples scored negative in both LAMP and ITS1 nested-PCR assays (Table 3). The sensitivity of the LAMP assay was determined to be 100% while its specificity is 76%. The 95% CI for specificity ranges from 59% to 92.5%.

**Table 3:** Performance of Mo2 LAMP on previously characterized *M*. *ozzardi* patient samples.

	Positive in Lima *et al*.^60^	Negative in Lima *et al*.^60^
LAMP Positive	51	8
LAMP Negative	0	25

## Discussion

Sensitive and specific tools are key to the success of neglected tropical disease control. Measuring changes in pathogen populations in both human and vector hosts are needed to ensure that interventions are achieving elimination goals, and to direct limited resources to areas of high or persistent prevalence. Monitoring of parasites in the blood/skin or in vectors by conventional techniques such as microscopy can be particularly difficult when prevalence is reduced due to control measures underway. A variety of immunoassays and DNA based methods are available for lymphatic filariasis, onchocerciasis and loiasis. However, for mansonelliasis only PCR assays mainly targeting ITS have been developed. To identify biomarkers with greater specificity and sensitivity, raw reads from sequencing data were analyzed for genomic repeat sequences. Highly repetitive DNA has been used in multiple diagnostic assays^45–47,56,57^. Using available genome sequence from several filarial parasites, we previously developed a multi-step bioinformatic pipeline which enabled the discovery of novel DNA biomarkers suitable for the development of a new diagnostic LAMP assay for *L*. *loa*^47^. Our goals were to apply this approach to mansonelliasis and identify repeat-based DNA biomarkers which can distinguish *Mansonella* parasites from other filarial species, as well as discriminate between the closely related *M*. *perstans* and *M*. *ozzardi*. Two *M*. *ozzardi* libraries (SRA run accession numbers: ERR126085 and ERR119619) have been sequenced by the Wellcome Trust Sanger Institute for the 50 Helminth Genomes Initiative (http://www.sanger.ac.uk/science/collaboration/50hgp). However, combined, these libraries totaled ~86 million reads equivalent to less than 1X coverage of the genome. It was estimated that 50X coverage of each genome was required to have sufficient data for repeat identification and assembly. Therefore, Illumina libraries were constructed and sequenced for both *M*. *perstans* and *M*. *ozzardi*. Our novel bioinformatic pipeline allowed us to identify species-specific repeats in two closely related *Mansonella* species without requiring the assembly of their genomes. Reads containing k-mers that were observed at a high-frequency and indicative of repeat sequence were assembled. After filtering against other filarial genomes, we identified candidate species-specific biomarkers for *M*. *perstans* and *M*. *ozzardi* of potential diagnostic value. Consensus repeat sequences with the highest number of aligned reads, which served as a proxy for copy number, were chosen as candidate biomarkers. Following further evaluation of consensus repeats by PCR and LAMP, Mp419 (*M*. *perstans*) and Mo2 (*M*. *ozzardi*), were selected as the best biomarker for each species. Subsequent analyses revealed that both repeat families are arranged in ladder arrays in their respective genomes. Mp419 is ~200 bp in length, whereas Mo2 is ~300 bp, and similar in size to other filarial repeat families used as biomarkers in various PCR- or LAMP-based diagnostic platforms namely *B*. *malayi Hha*I (322 bp), *W*. *bancrofti Ssp*I (195 bp), *L*. *loa* RF4 (440 bp), and *O*. *volvulus* O-150 (150 bp)^43^.

Both Mp419 and Mo2 repeats are most likely satellite DNA, characterized by non-protein coding, tandemly repeated sequences arranged head to tail in long arrays of up to 100 Mb. Satellite repeats can range from as little as 2 bps up to 1000 bps in length. Commonly however, as is the case here, the length of satellite monomers range from 150–180 bps or 300–360 bps, the length of DNA required to wrap around one or two nucleosomes^61^. The majority of satellite DNA is found in constitutive heterochromatin: those regions of the chromosomes that remain condensed throughout the cell cycle. Recent work suggests satellite DNA is actively involved in maintaining centromere structure, as well as in chromosome pairing and segregation^62^. As many repeats are non-coding and subject to fewer evolutionary constraints, their sequence can diverge even between closely related species^57^. Further, repeat sequences exist in genomes at high copy numbers and generally give better sensitivity in molecular diagnostic assays than single copy markers.

In addition to increasing specificity and sensitivity through the use of repeat sequences as biomarkers, further enhancements can be obtained in LAMP assays as the amplification reaction includes the use of four sequence-specific primers which anneal to six distinct regions within the target sequence^42^. A pair of loop primers are often added to accelerate the reaction^63^. Several parameters including primer design, reaction time and temperature, were optimized to ensure development of a robust assay. The analytical sensitivity of the colorimetric Mp419 LAMP assay was determined to be as little as 0.1 pg of *M*. *perstans* DNA within a 60 min reaction time, the equivalent of 1/1000^th^ of an mf. A similar level of sensitivity was observed using a previously published nested-PCR assay^31^ which requires ~4 hours to perform, 2 rounds of PCR and visualization by gel electrophoresis. The Mo2 LAMP assay has a limit of detection of 0.01 pg of *M*. *ozzardi* DNA or 1/10,000^th^ of an mf in 30 min. Both assays far exceed the theoretical limit of detection of one mf per ml obtained using conventional microscopy and concentration techniques^64^.

To determine if the LAMP assays are suitable for the evaluation of biological samples, a small number of samples from human (*M*. *perstans* and *M*. *ozzardi*) and insects (*M*. *perstans*) were examined. The Mp419 LAMP assay successfully detected *M*. *perstans* in human samples which were positive for mf by microscopic examination of blood smears and ITS1 nested-PCR. Although not evaluated in human samples from Latin America, we anticipate that the Mp419 assay would perform equally well in those areas since *M*. *perstans* collected from Africa and South America were found to be highly similar in phylogenetic analyses^3^. In insects, LAMP and nested-PCR showed good concordance except for 4 midge samples that were positive by nested-PCR but not LAMP. One possible explanation for the difference could be the detection of other *Mansonella* species in these insects by nested-PCR. Multiple species of *Mansonella* have been identified in Africa including *M*. *streptocerca*, *M. rodhaini*, *Mansonella vanhoofi*, *Mansonella leopoldi* and *Mansonella lopeenis*^7,24,65^. For such closely related species, nested-PCR can be problematic^32–34,38,40^ as a slight change in amplification conditions can result in an assay becoming non species-specific^10,57^. Therefore sequencing of the amplification product is required to discriminate between closely related species^30^. Upon sequencing the ITS1 products from the LAMP negative midge samples, all four exhibited more than 99% sequence identity with *M*. *perstans* ITS1. Another possible explanation for the observed difference could be variation in assay performance around the limit of detection in midge samples, as an individual midge is expected to carry a low amount of parasite DNA, with at most two filarial worms if the parasite is able to establish successfully^9,66^. Analysis of larger numbers of insect samples will be necessary to explore this further.

The performance of the Mo2 LAMP assay was evaluated on previously characterized human samples collected from villages along the Purus River in northwestern Brazil^60^. Individuals considered positive based on microscopy or by ITS2-based qPCR, also scored positive in Mo2 LAMP. This LAMP assay identified 8 additional positives among those samples previously scored negative by microscopy and ITS2-based qPCR^60^. We confirmed these samples were true positives using ITS1 nested-PCR. We also demonstrated the utility of the Mo2 LAMP using a *M*. *ozzardi* isolate from Venezuela (data not shown), highlighting its applicability across a wider geographical range. In addition, the Mo2 repeat sequence was found in both *M*. *ozzardi* libraries (strain Amazonas) available through NCBI (SRA run accession numbers ERR126085 and ERR119619) by blastn analysis. The exquisite specificity of the Mp419 and Mo2 LAMP assays enable a more accurate determination of transmission of *M*. *perstans* and *M*. *ozzardi* in Africa and Latin America.

Although molecular based assays such as conventional PCR are capable of detecting low-density infections, they are not suitable for large scale community surveillance due to complex procedures, expensive reagents and the requirement for specialized equipment. The operational simplicity of the LAMP technique makes it particularly appealing for neglected tropical diseases. Since many of these diseases are co-endemic, it is desirable to leverage resources and integrate diagnostic platforms wherever possible. We have previously developed DNA-based LAMP diagnostic assays specific for *B*. *malayi*^46^*, B*. *timori*^46^, *W*. *bancrofti*^45,53^, *O*. *volvulus*^51,56^, and *L*. *loa*^47^. With the addition of simple colorimetric LAMP assays for *M*. *perstans* and *M*. *ozzardi*, a single diagnostic platform is now available for all major filarial diseases which is suited to both human and insect use. These assays will greatly facilitate surveillance of insect vectors to determine transmission of *Mansonella* spp. parasites, which is currently performed by dissection and microscopy^67^.

In summary, mansonelliasis is considered one of the most neglected tropical diseases and is likely underdiagnosed as accurate tools for parasite detection are lacking. This study outlines the use of subtractive genome analyses for *in silico* identification of new biomarkers with remarkable specificity and development of rapid diagnostic tests for *M*. *perstans* and M. *ozzardi*. The tests offer significant improvement over current methods of *Mansonella* detection including specificity, sensitivity, speed and ease of use, as well as the option to perform tests on-site on both human and insect samples. While there are currently no filariasis control programs targeting *Mansonella* infection^68^, these new tests hold much promise in supporting control efforts in resource-limited settings.

## Methods

### Ethics statement

All research was approved by the appropriate committee, performed in accordance with all relevant guidelines/regulations and informed consent was obtained from all participants/or their legal guardians.

For *M*. *perstans*, An ethical clearance was obtained from the National Institutional Review board, Yaounde (REF: N° 2015/09/639/CE/CNERSH/SP) and administrative clearance from the Delegation of Public Health, South-West region of Cameroon (Re: R11/MINSANTE/SWR/ RDPH/PS/259/382). Approval for the study was granted by the “National Ethics Committee of Research for Human Health” in Cameroon. Special consideration was taken to minimize the health risks to which any participant in this study was exposed. The objectives of the study were explained to the consenting donor after which they signed an informed consent form. The participant’s documents were given a code to protect the privacy of the study subject. At the end of the study, the donor received a cure of mebendazole (100 mg twice a day for 30 days) against the infection of *M*. *perstans*^2^.

For *M*. *ozzardi*, study protocols were approved by the Institutional Review Board of the Institute of Biomedical Sciences, University of São Paulo, Brazil (1133/CEP, 2013). Written informed consent was obtained from all patients or their parents or guardians, if participants were minors aged <18 years. Diagnosed infections were treated with a single dose of 0.2 mg/kg of ivermectin after blood sampling^19^.

### Biological materials

Human blood samples with (n = 9) or without (n = 1) *M*. *perstans* mf as determined by microscopy were obtained from consenting donors in Cameroon as described previously^16^. Blood from non-endemic individuals (n = 5) residing in the U.S. was purchased (Quadrant Health Strategies, USA) for use as negative controls in nested PCR and LAMP assays.

*Culicoides milnei* midges were collected following feeding on a human volunteer positive for *M*. *perstans* as previously described^9^. Briefly, the volunteer sat under a drop trap in the lowered position. The trap was raised for 10–15 min to allow contact with wild midges then lowered to capture any attracted flies. After about 12 min, the expected time for trapped midges to be fully engorged, the flies were gently aspirated into 50 ml tubes. To obtain unfed *Culicoides*, females seeking a blood meal were aspirated into tubes as soon as they landed on collectors wearing protective clothing^9^. Species identification of adult wild caught *Culicoides* was done by microscopic examination of wing pigmentation pattern. Additional morphological characteristics including maxillary palps, interocular space and male genitalia were examined when wing pigmentation patterns proved inconclusive^9^. Laboratory reared, uninfected *Culicoides sonorensis* midges were obtained from Lee Cohnstaedt (Agricultural Research Service, USDA).

### DNA samples

Genomic DNA samples were generously donated by the following: *O*. *volvulus*, A. Alhassan (New England Biolabs) and *L*. *loa*, B.L. Makepeace and C. Hartley (University of Liverpool). WGA DNA from *W*. *bancrofti* was obtained from the NIH/NIAID Filariasis Research Reagent Resource Center (http://www.filariasiscenter.org). A previously published set of DNA samples from humans positive for *M*. *ozzardi* (n = 51) and humans negative for *M*. *ozzardi* (n = 31)^60^ were included in this study.

### DNA isolation and preparation

*M*. *perstans* genomic DNA from mf was prepared using a Genomic DNA Tissue MicroPrep kit (Zymo Research, USA) and the included protocol for hair and feathers, as directed by the manufacturer. DNA was extracted as above from *Culicoides sonorensis* midges as well as from individual unfed (n = 36) and fed (n = 36) *Culicoides milnei* midges. DNA was prepared from non-endemic whole blood samples using the Monarch Genomic DNA Purification Kit (New England Biolabs) as directed by the manufacturer. These genomic DNAs were used in all LAMP and nested PCR assays. Whole genome amplified (WGA) DNA was prepared as previously described^47^ from 4 individual midge samples for confirmatory experiments. Following purification, DNA quantity was determined using a Qubit dsDNA HS Assay kit in conjunction with a Qubit 2.0 Fluorometer as directed by the manufacturer (Life Technologies, USA).

### Illumina library construction and sequencing

The NEBNext Microbiome DNA enrichment kit was used as directed (New England Biolabs Inc., USA) to enrich *Mansonella* DNA and reduce human DNA contamination prior to library construction. The Illumina libraries were constructed for *M*. *perstans* and *M*. *ozzardi* using the NEBNext Ultra II FS DNA Library Prep Kit (New England Biolabs Inc., USA) as described by the manufacturer. For *M*. *perstans*, 2 ng of DNA was fragmented with the FS enzyme mix for 10, 20 or 30 minutes, and for *M*. *ozzardi*, 50 ng of DNA was fragmented for 5, 10 or 20 minutes. A library was prepared from each time point then PCR amplified with a different index primer to enable multiplexing. The quality and concentration of each library was determined using a 2100 Bioanalyzer with a high sensitivity DNA chip (Agilent Technologies, USA). Libraries were diluted to 4 nM with 10 mM Tris, 0.1 mM EDTA pH 8, and equal volumes of the 3 libraries from each species were mixed to create a pooled sample. Phi X DNA was added to balance base pair composition in these A:T rich filarial libraries prior to sequencing on a NextSeq500 platform (paired end, 150 bps).

### Bioinformatic analysis

Adapter trimming and removal of low-quality reads (phred score <20) was performed using the skewer program^69^. Reads originating from the phiX control or human genome (grch38 version GCF_000001405.36) were removed using the BBMap package, version 37.17 (https://sourceforge.net/projects/bbmap). K-mers of 20 nt in length were generated from the remaining *Mansonella* reads, using the jellyfish software^70^. Reads containing k-mers observed at high-frequency (frequency range 1,000 to 100,000) were selected using custom Perl scripts and were assembled into candidate repeat sequences using the Trinity assembler^71^. The candidate repeat sequences which had a blastn hit in any of the following genomes: *Homo sapiens*, *B*. *malayi*, *L*. *loa*, *O*. *volvulus*, *W*. *bancrofti*, (bit-score >=50, e-value <=1e-10), were removed, leaving behind candidate repeats specific to *Mansonella*. To ensure species-specificity, reads from each of the *Mansonella* species were mapped to the assembled repeats from its sister species using BBMap (mapping quality >=20). For each species, only the repeats which did not have any mapped reads from its sister species were retained as species-specific repeats. To achieve high sensitivity, only the repeat sequences with at least 50X coverage in the Trinity assembly were selected for further validation. The consensus repeats identified were then ranked by size (>300 bps) and % GC content to facilitate LAMP primer design^47^ (Fig. 1).

### Primer design

PCR primers were manually designed to characterize the genomic organization of the *Mansonella* repeats targeted by LAMP. The forward and reverse primer sequences for the *M*. *perstans* repeat Mp419 are (5′ ACAGTTGATTATTTGAAGGTGCTG 3′) and (5′ ACAATGATTATTTCTAAAGAATC 3′), respectively. The forward and reverse primer sequences for the *M*. *ozzardi* consensus repeat Mo2 are (5′ CTTGCGCGTAGSATTAGATCC 3′) and (5′ CGCAAACAGAAGCCYAAAWC 3′), respectively, where S = C or G, Y = C or T and W = A or T. The *M*. *perstans* LAMP primer set (Fig. 2) was designed manually using “A guide to LAMP primer design” available from the Eiken Chemical Co. (https://primerexplorer.jp/e/v4_manual/pdf/PrimerExplorerV4_Manual_1.pdf). The *M*. *ozzardi* consensus sequence was submitted to PrimerExplorer V5 (http://primerexplorer.jp/e/) generating primer sequences for F3, FIP, BIP, B3 and Loop primers (Fig. 3). Primers were synthesized (PCR and LAMP) and HPLC purified by Integrated DNA Technologies, USA.

### PCR assays

Genomic DNA was mixed with 12.5 μl of OneTaq Hot Start Quick-Load 2X Master Mix with standard buffer (New England Biolabs Inc., USA) containing 0.2 μM each of the forward and reverse primers in a final volume of 25 μl. Reactions were denatured once at 94 °C for 30 sec then cycled 36 times at 94 °C for 30 sec, 49 °C (*M*. *ozzardi*) or 45 °C (*M*. *perstans*) for 30 sec and 68 °C for 1 min followed by a final 5 min extension at 68 °C.

As a positive control for the presence of intact DNA, a 244 bp actin fragment was PCR amplified from 1 ng of various DNAs using OneTaq Hot Start Quick-Load 2X Master Mix with Standard Buffer (New England Biolabs Inc., USA) in 25 μl reactions supplemented with 3.5 mM MgCl_2_ and 0.2 μM each of the forward and reverse actin primers, and amplified as described previously^46^.

Amplification of the internal transcribed spacer 1 (ITS1) region was performed by nested PCR essentially as described^31^. The first PCR was initiated by mixing 2 μl of DNA with 12.5 μl of Quick-Load Taq 2X Master Mix (New England Biolabs Inc., USA) containing 0.2 μM each of ITS1-F and ITS1-R primers in a final volume of 25 μl. Reactions were denatured once at 95 °C for 30 sec then cycled 35 times at 95 °C for 30 sec, 58 °C for 30 sec and 68 °C for 45 sec followed by a final 5 min extension at 68 °C. For the second round of PCR, 2 μl of the initial PCR was diluted 1/250 in H_2_O. Two μl of each dilution was then added to reactions containing 0.2 μM each of the MpF1 and MpR1 primers and 12.5 μl Quick-Load Taq 2X Master Mix in a final volume of 25 μl. The reactions were amplified as described for the first PCR except the annealing temperature was set to 45 °C.

PCR reaction products were analyzed by electrophoresis on 1.5% agarose gels equilibrated with 1X Tris-borate/EDTA buffer. For 4 midge samples, WGA DNA was PCR amplified as described above for the first step of the nested PCR protocol using the ITS1-F and ITS1-R primers. The resulting 500 bp band was gel purified using the Monarch DNA Gel Extraction Kit (New England Biolabs) as described by the manufacturer then dideoxy sequenced on an Applied Biosystems 3730xl DNA Analyzer in both directions using the ITS1-F and ITS1-R primers.

### LAMP assays

LAMP reactions contained 1.6 μM each of primers FIP and BIP, 0.2 μM each of F3 and B3, 0.4 μM each of LF and LB, 12.5 μl of WarmStart Colorimetric LAMP 2X Master Mix (New England Biolabs Inc., USA) with 2 ul of template DNA, or H_2_O for non-template controls (NTCs) in a total volume of 25 μl. Reactions were incubated at the optimal temperature of 63 °C for 20–30 min (*M*. *ozzardi*) or 60 minutes (*M*. *perstans*) in a T100 Thermal Cycler (Bio-Rad Laboratories, USA). When a qPCR machine (CFX-96 Touch Thermal Cycler, Bio-Rad Laboratories, USA) was used to enable reaction dynamics to be monitored in real time, colorimetric reactions also contained 1X LAMP Fluorescent Dye (New England Biolabs Inc., USA) and were incubated at 63 °C for ~30 min (*M*. *ozzardi*: 36 cycles) or ~1 hr (*M*. *perstans*: 72 cycles) with a plate read step every 42 seconds. To record color changes, samples were scanned using an Epson Perfection v700 photo flatbed scanner (Epson America, Inc., USA).

### Statistical analysis

For determining the sensitivity and specificity of the LAMP assays on biological samples, the standard methods employing 2 × 2 contingency tables for evaluation of diagnostic tests were used^72^. When evaluating Mp419 LAMP on insect samples, a single assay namely ITS1 nested-PCR was used as the “gold-standard”. For human blood samples, LAMP was compared against two assays namely microscopy and ITS1 nested-PCR, which necessitated the use of the composite reference standard (CRS) test^72^ for statistical analysis. In this analysis, microscopy evaluations were used as the “imperfect gold standard” while the ITS1 nested-PCR assay was used as the “imperfect resolver”. In the case of the *M*. *ozzardi* Mo2 LAMP assay, previously characterized human blood samples^60^ were used as the “gold standard”.

The sensitivity of LAMP was calculated as the ratio of true-positives (positive in both the reference test and in LAMP) to the sum of true-positives and false-negatives (positive in the reference test but negative in LAMP). The specificity of the LAMP assay was calculated as the ratio of true-negatives (negative in both the reference test and the LAMP assay) to the sum of true-negatives and false-positives (negative in the reference test but positive in LAMP). The 95% confidence intervals for all sensitivity and specificity values were calculated in R, as previously described^72^.

## Supplementary information

Supplementary Information
