# Supplementary material for: In Silico Identification of Novel Biomarkers and Development of New Rapid Diagnostic Tests for the Filarial Parasites Mansonella perstans and Mansonella ozzardi
Source: Sci Rep. 2019 Jul 16;9:10275. doi: 10.1038/s41598-019-46550-9 (PMC6635353; doi:10.1038/s41598-019-46550-9)
Supplement: Supplementary file 1 — Supplementary Information [file 41598_2019_46550_MOESM1_ESM.pdf]

In Silico Identification of Novel Biomarkers and Development of New Rapid Diagnostic Tests for the Filarial Parasites *Mansonella perstans* and *Mansonella ozzardi*

Poole, C.B.[1], Sinha A.[1], Ettwiller, L.[1], Apone, L. [1], McKay, K. [1], Panchapakesa, V. [1], Lima N.F. [2], Ferreira M.U.[2], Wanji S.[3], and Carlow, C.K.S[1*]

[1]New England Biolabs, USA; Department of Parasitology, Institute of Biomedical Sciences, University of Sao Paulo, Brazil[2]; Department of Microbiology and Parasitology, University of Buea, Cameroon[3]

*Correspondence to carlow@neb.com

Supplementary Fig. 1

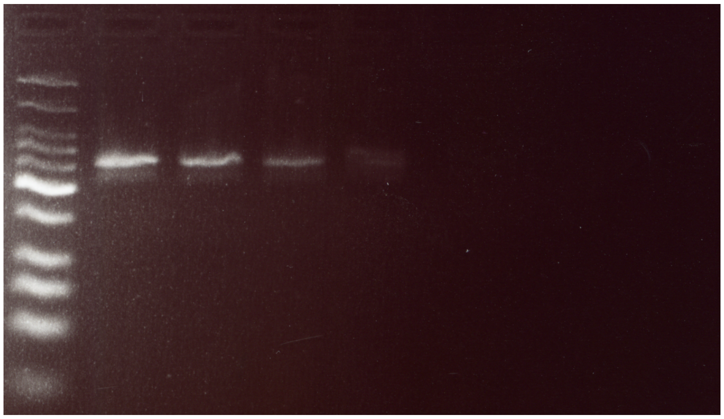

Supplementary Fig. 2A

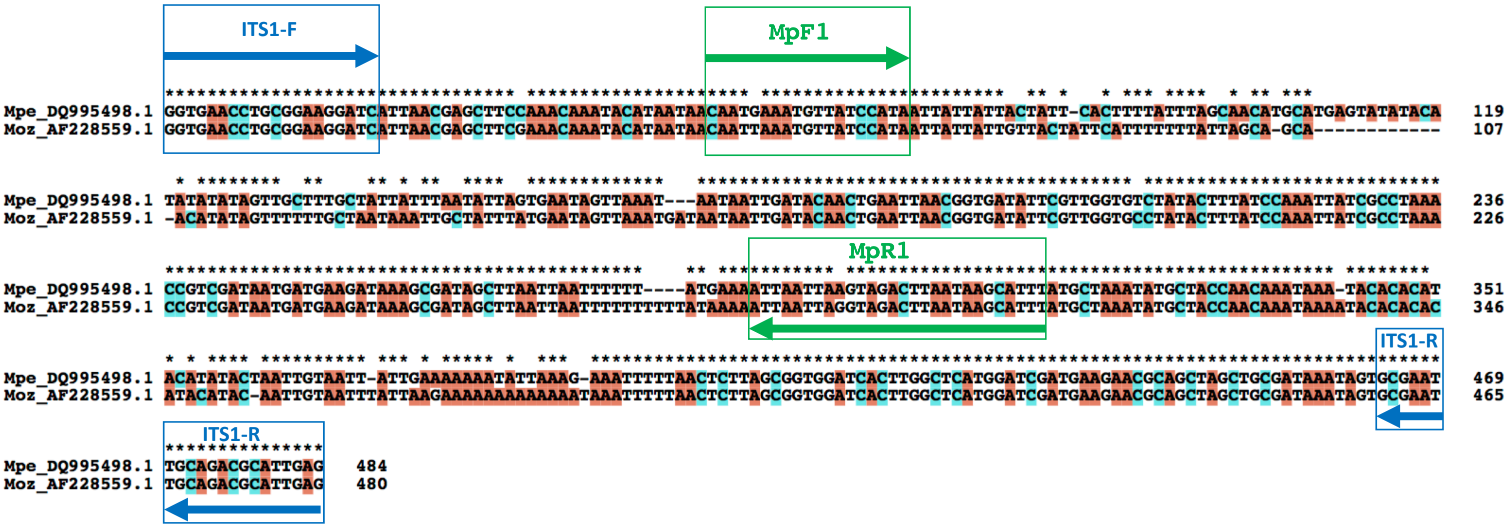

Supplementary Fig. 2B

```
                         ******************************************************************** *******************************************
midge_29AA  -------------AAGGATCATTAACGAGCTTCCAAACAAATACATAATAACAATGAAATGTTATCCATAATTATTATTACCATTCACTTTTATTTAGCAACATGCATGAGTATATACATATATA   125
midge_29L   -------------AAGGATCATTAACGAGCTTCCAAACAAATACATAATAACAATGAAATGTTATCCATAATTATTATTACTATTCACTTTTATTTAGCAACATGCATGAGTATATACATATATA   125
DQ995498.1  GGTGAACCTGCGGAAGGATCATTAACGAGCTTCCAAACAAATACATAATAACAATGAAATGTTATCCATAATTATTATTACTATTCACTTTTATTTAGCAACATGCATGAGTATATACATATATA   125
midge_29A   -----------GGAAGGATCATTAACGAGCTTCCAAACAAATACATAATAACAATGAAATGTTATCCATAATTATTATTACTATTCACTTTTATTTAGCAACATGCATGAGTATATACATATATA   125
midge_29Q   ----------NGGAAGGATCATTAACGAGCTTCCAAACAAATACATAATAACAATGAAATGTTATCCATAATTATTATTACTATTCACTTTTATTTAGCAACATGCATGAGTATATACATATATA   125

            *****************************************************************************************************************************
midge_29AA  TAGTTGCTTTGCTATTATTTAATATTAGTGAATAGTTAAATAATAATTGATACAACTGAATTAACGGTGATATTCGTTGGTGTCTATACTTTATCCAAATTATCGCCTAAACCGTCGATAATGAT   250
midge_29L   TAGTTGCTTTGCTATTATTTAATATTAGTGAATAGTTAAATAATAATTGATACAACTGAATTAACGGTGATATTCGTTGGTGTCTATACTTTATCCAAATTATCGCCTAAACCGTCGATAATGAT   250
DQ995498.1  TAGTTGCTTTGCTATTATTTAATATTAGTGAATAGTTAAATAATAATTGATACAACTGAATTAACGGTGATATTCGTTGGTGTCTATACTTTATCCAAATTATCGCCTAAACCGTCGATAATGAT   250
midge_29A   TAGTTGCTTTGCTATTATTTAATATTAGTGAATAGTTAAATAATAATTGATACAACTGAATTAACGGTGATATTCGTTGGTGTCTATACTTTATCCAAATTATCGCCTAAACCGTCGATAATGAT   250
midge_29Q   TAGTTGCTTTGCTATTATTTAATATTAGTGAATAGTTAAATAATAATTGATACAACTGAATTAACGGTGATATTCGTTGGTGTCTATACTTTATCCAAATTATCGCCTAAACCGTCGATAATGAT   250

            *** *************************************************************************************************************************
midge_29AA  GAAGATAAAGCGATAGCTTAATTAATTTTTTATGAAAATTAATTAAGTAGACTTAATAAGCATTTATGCTAAATATGCTACCAACAAATAAATACACACATACATATACTAATTGTAATTATTGA   375
midge_29L   GAAAATAAAGCGATAGCTTAATTAATTTTTTATGAAAATTAATTAAGTAGACTTAATAAGCATTTATGCTAAATATGCTACCAACAAATAAATACACACATACATATACTAATTGTAATTATTGA   375
DQ995498.1  GAAGATAAAGCGATAGCTTAATTAATTTTTTATGAAAATTAATTAAGTAGACTTAATAAGCATTTATGCTAAATATGCTACCAACAAATAAATACACACATACATATACTAATTGTAATTATTGA   375
midge_29A   GAAGATAAAGCGATAGCTTAATTAATTTTTTATGAAAATTAATTAAGTAGACTTAATAAGCATTTATGCTAAATATGCTACCAACAAATAAATACACACATACATATACTAATTGTAATTATTGA   375
midge_29Q   GAAGATAAAGCGATAGCTTAATTAATTTTTTATGAAAATTAATTAAGTAGACTTAATAAGCATTTATGCTAAATATGCTACCAACAAATAAATACACACATACATATACTAATTGTAATTATTGA   375

            ****** ********************************************** *****************************************
midge_29AA  AAAAAATATTAAAGAAATTTTTAACTCTTAGCGGTGGATCACTTGGCTCATGGATCGATGAAGAACGCAGCTAGCTGCGATAAATAGTGCGAATTG-------------   484
midge_29L   AAAAAATATTAAAGAAATTTTTAACTCTTAGCGGTGGATCACTTGGCTCATGGGTCGATGAAGAACGCAGCTAGCTGCGATAAATAGTGCGAATTG-------------   484
DQ995498.1  AAAAAATATTAAAGAAATTTTTAACTCTTAGCGGTGGATCACTTGGCTCATGGATCGATGAAGAACGCAGCTAGCTGCGATAAATAGTGCGAATTGCAGACGCATTGAG   484
midge_29A   AAAAAATATTAAAGAAATTTTTAACTCTTAGCGGTGGATCACTTGGCTCATGGATCGATGAAGAACGCAGCTAGCTGCGATAAATAGTGCGAATT--------------   484
midge_29Q   AAAAAACATTAAAGAAATTTTTAACTCTTAGCGGTGGATCACTTGGCTCATGGATCGATGAAGAACGCAGCTAGCTGCGATAAATAGTGCGAATT--------------   484
```

Supplementary Fig. 3

(A)

(Stage 1)

		Microscopy results = Imperfect gold standard (S)		
		**S+**	**S-**	
LAMP assay = New test (N)	**N+**	9	1	**10**
	**N-**	0	10	**10**
		**9**	**11**	**20**

(Stage 2)

		ITS1 nested-PCR results = Imperfect Resolver (R)		
		**S+ S-,R+**	**S-,R-**	
LAMP assay = New test (N)	**N+**	9+1 = 10	1-1 = 0	**10**
	**N-**	0 + 0 = 0	10 – 0 = 10	**10**
		**10**	**10**	**20**

(B)

a = 9
b = 1
c = 0
d = 10

a' = 1
=> a + a' = 9 +1 = 10
=> b – a' = 1 - 1 = 0

c' = 0
=> c + c' = 0 + 0 = 0
=> d – c' = 10 - 0 = 10

Sensitivity = (a + a') / ((a+c) + (a' + c'))
= (9 + 1) / ((9+0) + (1 + 0)) = 10/10 = 100%

Specificity = (d – c') / ((b + d) – (a' + c'))
= (10 – 0) / ((1 + 10) – (1 + 0)) = 10/10 = 100%

Supplementary Fig. 4

A.

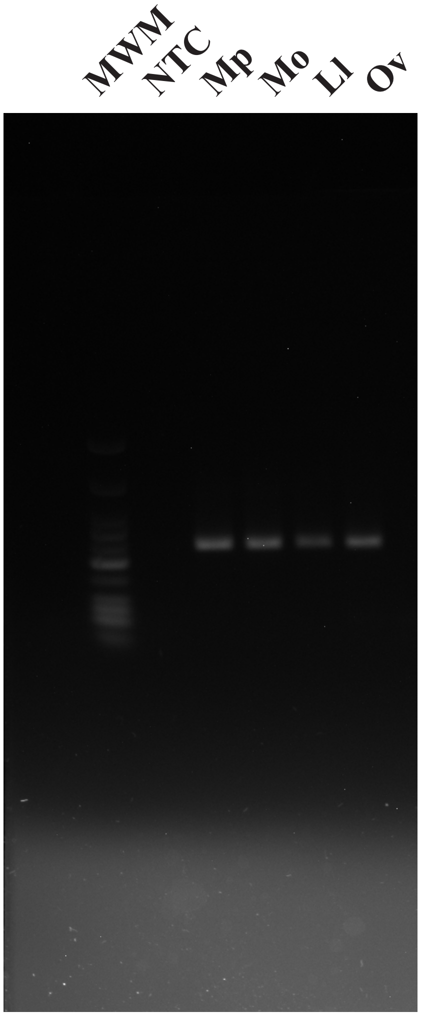

B.

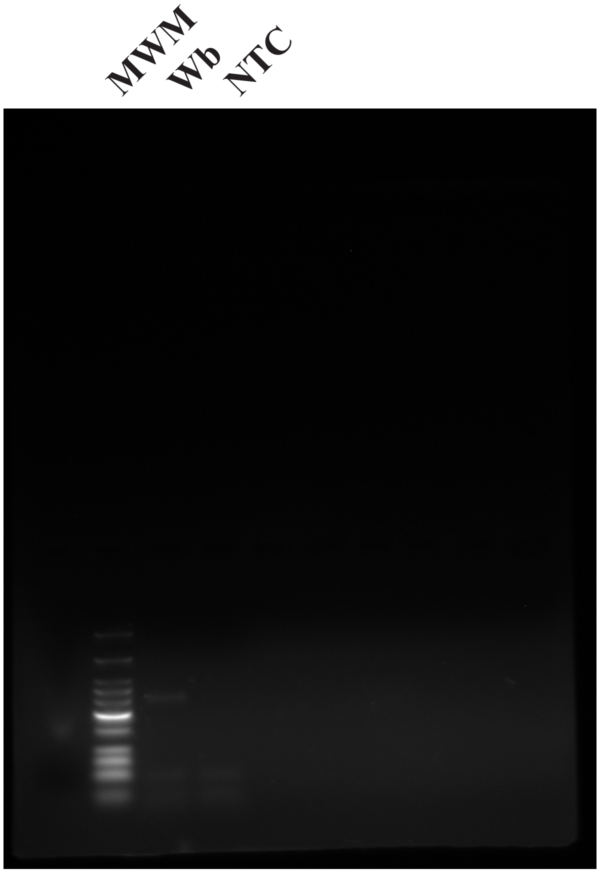

Supplementary Fig. 1. Sensitivity of *Mansonella* nested-PCR assay. *M. perstans* DNA ranging from 100-0.001 pg per reaction, was amplified by ITS1 nested PCR[31]. A negative (-) control containing 1 ng of HeLa DNA (New England Biolabs) was included. The Low Molecular Weight DNA ladder (New England Biolabs) was used as the MWM.

Supplementary Fig. 2. Sequencing is required to distinguish ITS1 amplicons of *M. perstans* from *M. ozzardi*. (A) Alignment of *M. perstans* (GenBank ID: DQ995498.1) and *M. ozzardi* (GenBank ID: AF228559.1) sequences shows that the published[31] primer sequences ITS1-F and ITS1-R (blue arrows), and MpF1 and MpR1 (green arrows) can amplify the ITS1 region of both species necessitating sequencing to correctly identify the species. (B) Multiple sequence alignment showing > 99.5 % identity between the reference ITS1 sequence from *M. perstans* (GenBank ID DQ995498.1) and the ITS1 region sequenced from the 4 midge samples that tested positive by ITS1 nested-PCR but negative by Mp419 colorimetric LAMP.

Supplementary Fig. 3. The two-stages of the composite reference standard (CRS) test for evaluating the performance of Mp419 LAMP assay on human samples. (A) In this CRS test, the LAMP assay is the new assay (N), microscopy is designated as the "imperfect standard" (S), and ITS1 nested-PCR is designated as the "imperfect resolver" (R). (B) Calculations of sensitivity and specificity. The changes introduced by R are a' and c'. For more details on methodology and terminology, see Hess *et al*.[72]

Supplementary Fig. 4. The original uncropped gels from which Fig. 4C was derived. The actin amplicons for *M. perstans* (Mp), *M. ozzardi* (Mo), *L. loa* (Ll), *O. volvulus* (Ov) and *W. bancrofti* (Wb) are shown in parts A and B respectively. The low molecular weight DNA ladder (New

England Biolabs) was used as the molecular weight marker (MWM). Water was substituted for DNA in the non-template controls (NTC).
